# Supplementary material for: Prevalence of physical, psychological and sexual intimate partner violence among women of reproductive age during COVID-19 in Ethiopia: a systematic review and meta-analysis
Source: PeerJ. 2024 Aug 23;12:e17812. doi: 10.7717/peerj.17812 (PMC11348896; doi:10.7717/peerj.17812)
Supplement: Supplemental Information 2 [file peerj-12-17812-s002.docx]

**The rationale for conducting the systematic review and meta-analysis**

Due to COVID-19 preventive measures such as stay at home and isolation leads to various violence against women. Intimate partner violence is one of the common violence during this pandemic in Ethiopia. However, no study was done in a comprehensive way on the prevalence of intimate partner violence among reproductive age women in Ethiopia during COVID-19. Thus, this systematic review and meta-analysis intended to provide the pooled prevalence of physical, psychological and sexual intimate partner violence among reproductive age women during COVID-19 in Ethiopia.

**The contribution that it makes to knowledge in light of previously published related reports, including other meta-analyses and systematic reviews.**

To our knowledge this is the first systematic review and meta-analysis to report the pooled prevalence of physical, psychological, and sexual intimate partner violence among reprodutive age women during COVID-19 in Ethiopia. Women in Ethiopia were affected by intimate partner violence during the pandemic, and physical, psychological and sexual intimate partner violence prevalence was high. Studies suports that intimate partner violence is a major public health issue affecting one out of every three pregnant women in Ethiopia. Intimate partner violence is critical problem that is occurring all over the world for many years now, but this condition has been increased during the lockdown situation of COVID-19.
